# Homothallic or Heterothallic? A Genomic Investigation Into the Sexual Capabilities of the Ascomycete Fungus *Clonostachys rosea*

**DOI:** 10.1111/mec.70486

**Published:** 2026-07-25

**Authors:** David Manyara, Andi M. Wilson, Edoardo Piombo, Mikael Brandström Durling, Martin Broberg, Birgit Jensen, Alessandra Ruffino, Sidhant Chaudhary, Henrik H. De Fine Licht, Mukesh Dubey, Magnus Karlsson

**Affiliations:** ^1^ Department of Forest Mycology and Plant Pathology Swedish University of Agricultural Sciences Uppsala Sweden; ^2^ Department of Plant and Environmental Sciences University of Copenhagen Copenhagen Denmark; ^3^ Forestry & Agricultural Biotechnology Institute, Department of Biochemistry, Genetics and Microbiology University of Pretoria Pretoria South Africa; ^4^ Department of Plant Physiology Umeå University Umeå Sweden; ^5^ Umeå Plant Science Center Umeå Sweden; ^6^ Department of Biotechnology and Biomedicine (DTU Bioengineering) Technical University of Denmark Kongens Lyngby Denmark

**Keywords:** *Clonostachys rosea*, evolution, heterothallism, homothallism, mating‐type locus, population genomics

## Abstract

Modes of reproduction and sexual strategies strongly influence the genetic diversity and evolutionary potential of a species. The ascomycete fungus *Clonostachys rosea* is reported to be homothallic (sexually self‐fertile), although a rapid decay of genome‐wide linkage disequilibrium is also reported, something that is not in line with an obligate homothallic mode of reproduction. To investigate this phenomenon, we identified the mating‐type (*MAT1*) locus in 66 genome‐sequenced *C. rosea* strains under the hypothesis that each strain contains genes from both *MAT1* idiomorphs. Eleven strains indeed contained genes from both the *MAT1‐1* and *MAT1‐2* idiomorphs, suggesting homothallism. However, most strains harboured genes from either the *MAT1‐1* or *MAT1‐2* idiomorphs and co‐existed in North America, Europe and China, suggesting heterothallism. The *MAT1* locus of heterothallic strains was highly conserved, and the linkage disequilibrium half decay distance was 1050 bp, suggesting sexual outcrossing. The presence of conserved *MAT1‐1* or *MAT1‐2* idiomorphs in strains of other *Clonostachys* species shows that heterothallism is likely the ancestral state. A phylogenetic analysis of 2800 single‐copy orthologous genes revealed that homothallic and heterothallic strains separated in two well‐supported clades, indicating a single lineage of homothallic *C. rosea* , likely originating in South America, followed by intercontinental dispersal. Homothallic *C. rosea* strains displayed higher nucleotide diversity than heterothallic strains, indicating a lack of outcrossing. This unique case of both homothallic and heterothallic lineages within the same species provides an opportunity to study the genomic consequences of selfing in very closely related strains.

## 1 Introduction

Many fungal species can reproduce both asexually and sexually during their life cycles (Nieuwenhuis and James 2016). Fungi that are capable of sexual reproduction can typically be described as either heterothallic or homothallic, depending on the precise way they undergo sexual reproduction (reviewed in Wilson et al. 2021). In heterothallic fungi, mating takes place when there is a physical interaction between two compatible individuals that are of the opposite mating type. In contrast, homothallic fungi can complete the sexual cycle in isolation. The difference in these sexual strategies is usually determined genetically by genes present at the mating‐type (*MAT1*) locus (Lin and Heitman 2007).

Fungal species exhibit a diversity of sexual strategies. While some species are obligately heterothallic, others may undergo mating‐type switching to generate suitable partners, as described in several yeast species including *Saccharomyces cerevisiae* (Becerra‐Rodríguez et al. 2026). Still others may entirely forgo the need for a partner by engaging in unisexual reproduction or primary homothallism. The *MAT1* locus regulates sexual reproduction in filamentous ascomycetes and controls partner recognition, meiosis, and other mating‐related processes. In heterothallic fungi, compatible individuals harbour alternate copies of the *MAT1* locus, termed idiomorphs (Metzenberg and Glass 1990). Individuals of the MAT1‐1 mating type harbour genes associated with the *MAT1‐1* idiomorph, while individuals of the MAT1‐2 mating type possess *MAT1‐2*‐associated genes. The completion of sexual reproduction typically requires the expression of genes from both the *MAT1‐1* and *MAT1‐2* idiomorphs, hence the requirement that heterothallic species physically interact ahead of sexual development (Ni et al. 2011). Homothallic species, on the other hand, typically harbour both *MAT1‐1‐* and *MAT1‐2‐*associated genes within a single genome and/or cell, allowing the expression of both types of genes and thus enabling independent sexual reproduction (Wilson et al. 2015).

Homothallic species tend to evolve from heterothallic ancestors (Yun et al. 1999). This occurs when a genetic event, such as unequal crossing over, combines genes from both *MAT1* idiomorphs within a single genome (Debuchy and Turgeon 1996). Because of this, the *MAT1* locus structure is often not conserved in homothallic fungi and can differ even among close relatives. In some cases, the alternate idiomorphs may even be found unlinked in the genome, resulting in two genomic regions with *MAT1* locus information (Rydholm et al. 2007). For example, self‐fertility has evolved independently a number of times in both *Neurospora* (Gioti et al. 2012) and *Aspergillus* (Ojeda‐López et al. 2018) with individual homothallic species harbouring different *MAT1* locus structures depending on the precise mechanisms that brought the *mat* genes together. These repeated transitions towards homothallism support the hypothesis of “reproductive assurance”, whereby species evolve mechanisms that ensure self‐compatibility and thereby facilitate sexual reproduction when compatible partners are rare or absent. While this concept has been studied primarily in yeasts (Nieuwenhuis and Immler 2016; Nieuwenhuis and James 2016; Nieuwenhuis et al. 2018), similar selective pressures may also influence transitions between sexual strategies in filamentous fungi. Transitions between sexual strategies are common across the fungal tree of life, with transitions towards homothallism generally occurring more frequently than transitions towards heterothallism (Wolfe and Butler 2022). While hypotheses based on reproductive assurance, genome renewal, DNA repair, and the production of environmentally resistant ascospores have been put forward, selection for homothallism is likely dependent on a combination of these factors. It may also be the case that lineage‐specific factors determine whether transitions between sexual strategies are more or less common.

The *mat* genes can be classified as primary and secondary genes (Wilson et al. 2021). The primary *mat* genes are the defining genes of each idiomorph and are almost universally present at the relevant idiomorph. The primary *mat* genes are *mat1‐1‐1* and *mat1‐2‐1*, both of which encode transcription factors (Ferreira et al. 1998; Klix et al. 2010). The MAT1‐1‐1 protein harbours an alpha‐box domain, and the MAT1‐2‐1 protein harbours an HMG‐box. Both genes have been functionally characterized in a variety of model and non‐model fungi and have proven to be essential for sexual development in almost all cases (Coppin et al. 1997; Ferreira et al. 1998; Glass et al. 1990; Klix et al. 2010). They both contribute to the initial formation of reproductive tissue, regulation of the pheromone system, and production of ascomata—the structures wherein the sexual ascospores are produced. The secondary *mat* genes (e.g., *mat1‐1‐2, mat1‐1‐3*) are less conserved and typically show lineage‐specific presence (Wilken et al. 2017). There is a paucity in functional data for these genes, and where this data does exist, there are species‐specific differences in their functions. For example, the *mat1‐1‐3* gene is entirely dispensable for sexual reproduction in *Sordaria macrospora* (Klix et al. 2010) but essential for fertility in *Fusarium graminearum* (Zheng et al. 2013).

The lack of outcrossing in obligate homothallic species is predicted to result in the accumulation of deleterious mutations, a process known as Muller's ratchet (Felsenstein 1974; Muller 1932, 1964). Over the long term, this continuous accumulation of harmful mutations can reduce fitness and increase extinction risk, especially in small or strictly selfing populations (Gioti et al. 2013; Loewe and Cutter 2008; Pamilo et al. 1987). Fungal systems with suppressed recombination around the *MAT1* locus show signatures consistent with relaxed selective constraint and mutation accumulation, including homothallic *Neurospora* species and anther‐smut fungi *Microbotryum*, where non‐recombining mating‐type regions exhibit degeneration such as frameshifts and premature stop codons in *mat* genes (Fontanillas et al. 2015; Wik et al. 2008). In the short term, however, obligate homothallism may allow the conservation of favourable combinations of alleles, providing high fitness to a homothallic, essentially clonal, lineage. Experimental work in the homothallic fungus *A. nidulans* has shown that selfed progeny can exhibit higher mean fitness than outcrossed progeny, despite reduced recombination (López‐Villavicencio et al. 2013), and similarly successful clonal or selfing lineages have been described among plant‐associated pathogens and mutualists, including *Phytophthora infestans* and *Epichloe* species (Drenth et al. 2019; McDonald and Linde 2002).

The fungus *Clonostachys rosea* (Schroers et al. 1999) is a filamentous ascomycete from the *Hypocreales* order. It is the anamorph of the teleomorph *Bionectria ochroleuca* (Schwein.) (Schroers and Samuels 1997; Schroers et al. 1999; Schroers 2001). *Clonostachys rosea* is the recommended species name under the one fungus, one name principle (Rossman 2014). Two variants of *C. rosea* can be found in the literature, *C. rosea* forma (f.) *rosea* and *C. rosea* f. *catenulata*, although they constitute a single species based on genealogical concordance phylogenetic species recognition (Moreira et al. 2016). The sexual fruiting body, the perithecium, is mostly found in pantropical and subtropical regions, while conidial (anamorph) strains of *C. rosea* are cosmopolitan (Schroers et al. 1999). *Clonostachys rosea* was reported to be homothallic, as strains originating from ascospores frequently produce perithecia in single cultures on rich media (Schroers et al. 1999). Notably, conidial strains only rarely form perithecia under the same conditions. However, a population genomic study of *C. rosea* (Broberg et al. 2018) reported rapid decay of genome‐wide linkage disequilibrium (LD), which is not expected given an obligate homothallic mode of reproduction. The closely related species *C. chloroleuca* does not produce perithecia in single culture and was reported as likely heterothallic (Moreira et al. 2016).

Strains of *C. rosea* and the closely related species *C. chloroleuca*, *C. farinosa* , *C. rhizophaga* and *C. solani* are typically isolated from a wide variety of ecological niches, including soil, other ascomycete fungi, plant litter and from living plants (Mendoza García et al. 2003; Mueller and Sinclair 1986; Nobre et al. 2005; Walker and Maude 1975), but rare isolations from nematodes and arthropods are also reported (Haarith et al. 2020; Verdejo‐Lucas et al. 2002). This broad habitat and cosmopolitan distribution of *Clonostachys* species can be correlated with an ecological generalist lifestyle, which includes plant endophytism, rhizosphere competence, polyphagous ability and mycoparasitism (Chatterton and Punja 2012; Li et al. 2002; Maillard et al. 2020; Saraiva et al. 2015; Shigo 1958). The broad nutritional versatility that characterizes generalist behaviour in *C. rosea* is suggested to be important for the ability of certain strains to control plant diseases and their commercial use as biological control agents (Jensen et al. 2022).

To shed light on the reproductive mode of *C. rosea* , we performed genome sequencing and identified the *MAT1* locus in 66 *C. rosea* strains under the hypothesis that each strain contains genes from both *MAT1* idiomorphs, as expected from the reported homothallism. Although 11 strains did indeed contain genes from both the *MAT1‐1* and *MAT1‐2* idiomorphs, most strains harboured either the *MAT1‐1‐* or *MAT1‐2‐*associated genes, suggesting heterothallism. A phylogenetic analysis of 2800 single‐copy orthologues revealed that homothallic and heterothallic strains clustered in two separate clades, indicating a single lineage of homothallic *C. rosea* , likely originating in South America, followed by inter‐continental dispersal. The presence of both homothallic and heterothallic lineages within a single species provides an excellent opportunity to study the genomic consequences of selfing.

## 2 Materials and Methods

### 2.1 Fungal Strains, Culture Conditions and DNA Extraction

*Clonostachys rosea* strains (Table S1 in Supporting Information S1) were revived from glycerol stocks stored at −70°C at the Department of Forest Mycology and Plant Pathology at the Swedish University of Agricultural Sciences, and maintained on potato dextrose agar (PDA, Oxoid, Cambridge, UK) at 25°C in darkness. For DNA extraction, strains were grown in 200 mL liquid Czapek‐Dox medium (Sigma‐Aldrich, Steinheim, Germany), Vogel's minimal medium (Vogel, 1956) or malt extract (1.75%) with peptone (0.25%) medium at room temperature, shaking at 120 rpm. Cultures were harvested after 3–13 days, depending on growth rate, by snap freezing in liquid nitrogen, and then freeze‐dried. High‐quality genomic DNA was extracted using a cetyltrimethylammonium bromide (CTAB)/chloroform‐based protocol or the Qiagen‐tip 100 kit (Qiagen, Hilden, Germany) as described previously (Broberg et al. 2018).

### 2.2 Genome Sequencing and Assembly

Base coverage for 10 *C. rosea* genomes was generated using Illumina HiSeqX paired‐end sequencing with a 350 bp insert size and 150 bp read length following standard library preparation kits. Sequence read data for these 10 *C. rosea* strains, together with reads from 52 previously sequenced *C. rosea* strains (Broberg et al. 2018), were assembled with ABySS ver. 1.3.6 (Simpson et al. 2009), as described by Broberg et al. (2018). Assembly completeness was assessed with Benchmarking Universal Single‐Copy Orthologs (BUSCO) v.5.1.2 (Seppey et al. 2019) using the “sordariomycetes odb12” lineage dataset, and scaffolds shorter than 500 bases were removed using the “funannotate sort” function in Funannotate v.1.8.15 (Palmer and Stajich 2020).

### 2.3 Genome Annotation and Gene Prediction

RNA‐seq data from *C. rosea* strains IK726, ACM941, and 88‐710 (Table S2 in Supporting Information S1) were retrieved and adapter‐ and quality‐trimmed using bbduk v.38.90 (Bushnell 2014). For samples generated from *C. rosea* grown in interaction with other organisms (e.g., plants or plant pathogens), trimmed reads were mapped to the *C. rosea* IK726 ver. 2 reference genome (GCA_902827195.2) (Broberg et al. 2018) using STAR v.2.7.9a (Dobin et al. 2013) with default parameters. Reads that did not align to the *C. rosea* genome were identified with “samtools view” v.1.19 (Danecek et al. 2021) and removed using “seqtk subseq” v.1.2‐r101 (Li 2021). Transcriptomes were then assembled with Trinity v.2.11.0 (Grabherr et al. 2011) using the option “‐‐jaccard_clip”.

Funannotate v.1.8.15 (Palmer and Stajich 2020) was used to filter and rename the scaffolds of the 62 newly assembled *C. rosea* genomes, together with four additional *C. rosea* genomes (strains ACM941, CanS41, IK726 and 88‐710), four *C. farinosa* genomes (synonym *C. byssicola*), four *C. chloroleuca* genomes, three *C. rhizophaga* genomes, one *C. solani* genome, and one *C*. sp. CBS 192.96 genome retrieved from the National Center for Biotechnology Information (NCBI) and Mycocosm (Table S3 in Supporting Information S1). All genomes underwent repeat identification with RepeatModeler v.2.0.1 (using the “LTRStruct” option) and repeat masking with RepeatMasker v.4.1.1 using default parameters (Flynn et al. 2020). Funannotate was trained for *C. rosea* gene prediction using the *C. rosea* IK726 ver. 2 reference genome (GCA_902827195.2) (Broberg et al. 2018) and the options “‐‐optimize_augustus ‐‐organism fungus –busco_db sordariomycetes”, incorporating Trinity‐assembled transcripts as transcript evidence and UniProt protein sequences obtained via “funannotate setup” as protein evidence. The trained gene model was then applied to predict genes in each newly assembled *C. rosea* genome using the same parameters except for “optimize_augustus”. Functional annotation of predicted proteins was performed with InterProScan v.5.48‐83.0 (Jones et al. 2014) using the options “‐‐iprlookup ‐‐goterms ‐‐pathways”. Secretome prediction followed the pipeline of Piombo et al. (2023). These annotations were integrated using “funannotate annotate” v.1.8.15 (Palmer and Stajich 2020), with the options “‐‐signalp ‐‐iprscan ‐‐busco_db sordariomycetes”.

Regarding nomenclature, we here use uppercase letters in italics for the *MAT1* locus and the *MAT1‐1*/*MAT1‐2* idiomorphs (Turgeon and Yoder 2000), while we use lowercase letters in italics for genes in *Clonostachys* (Jensen et al. 2022). The *MAT1* locus and corresponding *mat* genes from strains *C. rosea* CBS 100502 (*MAT1‐1*) and CBS 103.94 (*MAT1‐2*) were identified using the MAT1‐1‐1 and MAT1‐2‐1 proteins from *E. festucae* (NCBI accession numbers AKH05039.1 and AEI72619.1, respectively) and a local tBLASTn analysis. Up to 10 predicted proteins upstream and downstream of *mat1‐1‐1* and *mat1‐2‐1* were used in BLASTp similarity analyses against the NCBI nr database to determine the extent of the *MAT1* locus in these two strains. In both idiomorphs, the *MAT1* locus was flanked by homologues of APN2 and SLA2. These full length *MAT1‐1* and *MAT1‐2* loci were subsequently used in local BLASTn analyses against the remaining 77 genomes to determine the *mat* gene content and predict the mating type. To assess possible degeneration of homothallic *mat* genes, codon alignments were examined for premature stop codons and frameshift mutations, and selection analyses were conducted on *mat1‐2‐1* and *mat1‐2‐9* with *C. rhizophaga* YKD0085 as outgroup using HyPhy RELAX (Wertheim et al. 2015) and PAML codeml branch models (Yang 2007), with homothallic lineages defined as foreground branches. CLINKER as implemented in CAGECAT (Gilchrist and Chooi 2021) was used to generate synteny maps of the *MAT1* loci of selected strains. Mating type and sexual strategy was mapped onto a world map using mapPies in the R package rworldmap (South 2011).

### 2.4 Phylogenomic Analyses

The BUSCO proteins common to all 66 *C. rosea* genomes (62 newly assembled genomes and the four previously published *C. rosea* genomes from strains ACM941, CanS41, IK726 and 88‐710) and the 13 additional genomes from other *Clonostachys* species were aligned using MUSCLE ver. 5.1.0 (Edgar 2004) with default settings. The subsequent alignments were trimmed using trimAl ver. 1.1.4 (Capella‐Gutiérrez et al. 2009) and the ‐automated1 flag. Each alignment was used to infer single‐protein maximum likelihood trees using IQTree ver. 2.2.2.7 (Minh et al. 2020; Nguyen et al. 2015) with 1000 ultrafast replicates for bootstrapping (‐B 1000). A combined tree was then estimated using ASTRAL ver. 5.7.7 (Zhang et al. 2018), using local posterior probabilities (LPP) as a measure of branch and topological support. An LPP value close to 1 suggests high support for that node and that there is congruence between the individual gene trees for that node. The final tree was visualized in FigTree ver. 1.4.4. (http://tree.bio.ed.ac.uk/software/tree/) and edited in Affinity Designer 2.

### 2.5 Read Alignment, SNP Calling, and Quality Filtering

Illumina paired‐end reads from 63 *C. rosea* strains were analysed, comprising of the 62 genomes newly assembled in this study and the previously published reference strain *C. rosea* IK726 ver. 1 (Karlsson et al. 2015) (Table S1 in Supporting Information S1). Reads were adapter‐ and quality trimmed using bbduk v.38.90 (Bushnell 2014) with default settings. Trimming quality was assessed with fastQC v.0.11.9 (https://www.bioinformatics.babraham.ac.uk/projects/fastqc) and cleaned reads were mapped to the *C. rosea* IK726 ver. 2 genome (GCA_902827195.2) (Broberg et al. 2018) using Bowtie2 v.2.4.2 (Langmead and Salzberg 2012). Duplicate reads were identified with Picard MarkDuplicates v.2.18.29 (https://broadinstitute.github.io/picard) and BAM files with flagged duplicates were indexed using SAMtools v.1.19 (Danecek et al. 2021). Single nucleotide polymorphisms (SNPs) between the mapped reads and the *C. rosea* IK726 ver. 2 reference genome were called with BCFtools v.1.12 (Danecek and McCarthy 2017) using the parameters “‐excl‐flags UNMAP, SECONDARY, QCFAIL, DUP” for “bcftools mpileup” and “‐c ‐‐ploidy 1 ‐v ‐Oz” for “bcftools call”. Initial filtering of the resulting Variant Call Format (VCF) file was performed with VCFtools v.0.1.17 (Danecek et al. 2011) using the following criteria: “‐‐max‐missing 0.25, ‐‐min‐meanDP 40, ‐‐minQ 500, ‐‐minDP 10, ‐‐maf 0.05, ‐‐remove‐indels, and ‐‐recode”.

To minimize SNP detection errors, further quality filtering retained only bi‐allelic SNPs with sequencing depth between 55 and 92 (mean genome wide coverage 73.61). Based on the structure of the *MAT1* locus, the resulting dataset was then partitioned into homothallic and heterothallic strains for downstream analyses. Additional filtering was applied separately to each group: loci were required to have at least 20 reads, and strong allelic support within each strain was required, such that the called allele (reference or alternate) was supported by at least 90% of reads at that site. Loci not meeting these criteria were coded as missing data. Finally, SNPs with more than 25% missing data and a minor allele frequency (MAF) below 5% were removed from each dataset.

### 2.6 Population Structure Analyses

Genetic structure among the 63 *C. rosea* strains (Table S1 in Supporting Information S1) was analysed using principal component analysis (PCA) implemented in the R package SNPRelate v.1.4.0 (Zheng et al. 2012), and model‐based clustering in LEA v.3.18.0 (Frichot and François 2015). The analyses were performed on the original VCF file containing SNPs of all 63 strains, generated after SNP calling but prior to quality filtering. To reduce LD, SNPs were pruned with PLINK v.1.90b7 (Purcell et al. 2007) using a window size of 50 SNPs, a step size of 5 SNPs, and an LD threshold of 0.5. The dataset was further filtered to retain SNPs with coverage > 20, no missing data, and MAF > 10%. PCA was performed using the function snpgdsPCA in R v.4.4.2 (R Core Team 2025), and the first two eigenvectors were plotted to visualize population structure. Population structure was further assessed using sparse nonnegative matrix factorization (sNMF) (Frichot et al. 2014), which was used to estimate ancestry proportions and admixture levels for each strain. Twenty independent runs were carried out for *K* = 1–10, and the minimum cross‐entropy was observed at *K* = 6.

A phylogenetic network was also constructed to investigate reticulate evolutionary relationships among the 63 strains. For this purpose, the LD‐pruned and filtered VCF file was converted to NEXUS format using the vcf2phylip v.2.0 script (Ortiz 2019), and a neighbour‐joining phylogenetic network was then inferred in SplitsTree v.4.19.2 (Huson and Bryant 2006).

### 2.7 Population Genetic Analyses

Filtered SNP datasets for homothallic and heterothallic strains, generated as described in the previous section, were used for all population genetic analyses. Minor allele frequency spectra, Tajima's D, and nucleotide diversity (π) metrics were used to assess and quantify levels of variation between homothallic and heterothallic groups. Minor allele frequencies were calculated using the “‐‐freq” option in VCFtools v.0.1.17 (Danecek et al. 2011) and visualized with ggplot2 in R v.4.4.2 (R Core Team 2025). Tajima's D (Tajima 1989) and nucleotide diversity (Tajima 1983) were calculated separately for homothallic and heterothallic datasets in 10 kb sliding windows with 5 kb steps using the vcfR package v.1.14.0 (Knaus and Grünwald 2017). Differences in Tajima's D and nucleotide diversity between groups were assessed via 1000 bootstrap resamplings within each group, with mean values recalculated per iteration and compared using 95% confidence intervals and empirical *p* values (significant if *p* < 0.05 or *p* > 0.95).

Linkage disequilibrium was quantified from pairwise *r* ^2^ values (Hill and Robertson 1968) between SNPs using VCFtools v.0.1.17 (Danecek et al. 2011) with the “‐‐haploid” flag. Pairwise *r* ^2^ values were calculated within 10 kb windows (5 kb step size), and mean *r* ^2^ per window was used to assess LD decay. Physical distances between SNPs were binned into 100 bp intervals, and mean *r* ^2^ per bin was modelled with LOESS smoothing in R. The distance at which LD decayed to half its maximum was estimated as the midpoint of the bin where the LOESS curve crossed 50% of the maximum *r* ^2^. Ninety‐five percent confidence intervals were obtained by 1000 bootstrap resampling, and differences between homothallic and heterothallic groups were tested using a Wilcoxon rank‐sum test.

Shared and group‐specific SNPs were identified using BCFtools v.1.12 (Danecek and McCarthy 2017), where shared SNPs segregated in both groups and group‐specific SNPs occurred in only one group. Minor allele frequencies for each SNP set were calculated using VCFtools v.0.1.17 and visualized with ggplot2. To test for differences in nonsynonymous mutation accumulation associated with sexual strategy, nucleotide diversity was calculated separately for the predicted nonsense, missense, and synonymous SNPs in shared and group‐specific datasets. SNP functional impacts were predicted and annotated using SnpEff v.5.2e (Cingolani et al. 2012): predicted high‐impact (nonsense) SNPs are defined as non‐synonymous changes predicted to disrupt protein function via stop codon gain or loss; predicted moderate‐impact (missense) SNPs are non‐synonymous changes potentially altering protein sequence without disrupting function; and predicted low‐impact (silent) SNPs are generally synonymous and neutral. Statistical significance of nucleotide diversity differences was evaluated using 1000 bootstrap resamplings, with empirical *p* values defined as the proportion of iterations in which nucleotide diversity from one group exceeded that of the other (significant if *p* < 0.05 or *p* > 0.95).

To further examine selection pressures between homothallic and heterothallic groups, pN/pS ratios were calculated using all SNPs segregating within each group for homothallic and heterothallic groups. pN/pS ratios were calculated as the number of nonsynonymous SNPs per nonsynonymous site divided by the number of synonymous SNPs per synonymous site. Ratios above one indicate positive selection favouring protein changes, ratios below one suggest purifying selection against deleterious changes, and a ratio of one implies neutrality (Hurst 2002; Tanaka and Nei 1989). Ratios were computed for each gene with SNPs using a custom Python script (https://github.com/markhilt/mutation_analysis/blob/main/dnds.py), which counts potential synonymous and nonsynonymous sites per gene based on the reference, and the observed synonymous and nonsynonymous SNPs, assuming closely related strains and therefore no correction for multiple substitutions. Gene‐wise pN/pS ratios were then averaged within homothallic and heterothallic groups, and differences in selection levels between groups were tested using Welch's *t*‐test and Wilcoxon rank‐sum test in R v.4.4.2 (R Core Team 2025).

### 2.8 Phenotypic Analyses

The ability of homothallic strains to produce perithecia in single culture was investigated by inoculating strains on PDA (Oxoid, Cambridge, UK) in Petri dishes in duplicates, incubation at 20°C, followed by daily examination for 1 month using an Olympus SZD‐ILLD dissection microscope to observe mature perithecia. Release of asci and ascospores from perithecia was observed using an Olympos BX 60F5 microscope. Images were captured with a Canon DS 124491 camera. The ability of perithecia formation in crosses between selected MAT1‐1 and MAT1‐2 heterothallic strains was investigated by dual confrontation assays where 5 mm diameter agar plugs with actively growing mycelium from each strain were placed on PDA and synthetic crossing (SC) medium (Westergaard and Mitchell 1947) and incubated at 20°C in darkness for 1 month. Three biological replicates for each medium were included. Furthermore, dual confrontation assays between selected homothallic strains were performed in duplicates where 5 mm diameter agar plugs with actively growing mycelium from each strain were placed on PDA and incubated at 20°C in darkness for 1 month to assess self/non‐self‐recognition and hyphal fusion.

## 3 Results

### 3.1 Features of the *Clonostachys rosea* Genomes

The 62 *de novo* short‐read Illumina genome assemblies generated in this study had an average size of 57.96 Mb, ranging from 53.39 Mb ( *C. rosea* CBS 193.94) to 62.27 Mb ( *C. rosea* 1829) (Table S4 in Supporting Information S1). Genome assembly size was positively associated with repeat content (Spearman's *ρ* = 0.853, adjusted *p* = 1.11 × 10^−17^) and k‐mer‐based genome‐size estimates (Spearman's *ρ* = 0.429, adjusted *p* = 0.0061), indicating that variation in assembly size is associated with both repetitive DNA content and underlying differences in genome size among strains.

The predicted number of protein‐coding genes averaged 18,578 per genome, with a range of 17,638 ( *C. rosea* SHW‐3‐1) to 19,366 ( *C. rosea* 1829). Assemblies contained an average of 1133 scaffolds (range: 227 for *C. rosea* SHW‐3‐1 to 2369 for *C. rosea* CBS 649.80). Predicted gene number was positively associated with scaffold count (Spearman's *ρ* = 0.697, adjusted *p* = 8.44 × 10^−10^) and negatively associated with N50 (Spearman's *ρ* = −0.573, adjusted *p* = 2.51 × 10^−6^), suggesting that assembly fragmentation contributed to variation in gene prediction.

Genome completeness assessed using the “sordariomycetes odb12” BUSCO lineage dataset ranged from 88.3% to 98.7%, indicating near‐complete recovery of conserved genes (Table S4 in Supporting Information S1). BUSCO completeness increased with N50 (Spearman's *ρ* = 0.427, adjusted *p* = 7.65 × 10^−4^), suggesting that assembly contiguity contributed to variation in BUSCO recovery. A total of 2805 complete predicted BUSCO proteins were shared among all 79 *Clonostachys* genomes that were included in this study. Out of these, 5 were excluded as phylogenetically uninformative, leaving 2800 genes for downstream phylogenomic analyses.

### 3.2 Distribution of Mating‐Type Genes in *Clonostachys* Strains

**FIGURE 1:**
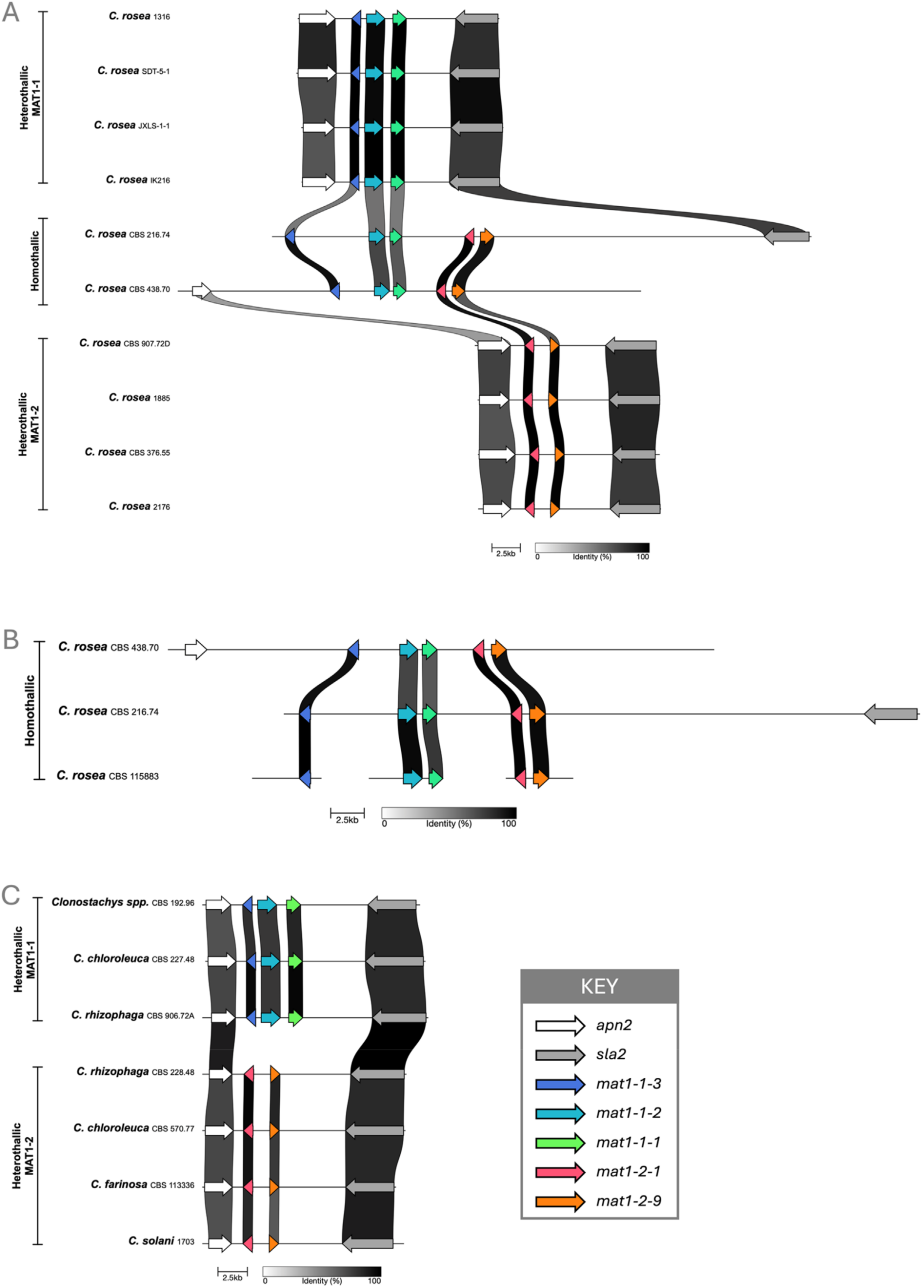
*MAT1* locus organization in *Clonostachys* (A) Synteny maps of the *MAT1* loci in selected heterothallic and homothallic *C. rosea* strains. The *MAT1* locus in *C. rosea* is flanked by *apn2* and *sla2*, a typical arrangement in other Pezizomycotina species. In heterothallic strains, the *MAT1‐1* idiomorph harbours the *mat1‐1‐1*, *mat1‐1‐2* and *mat1‐1‐3* genes, while the *MAT1‐2* idiomorph harbours the *mat1‐2‐1* and *mat1‐2‐9* genes. In homothallic strains, all five *mat* genes are found in an expanded locus, separated by highly repetitive sequence. (B) Synteny maps of the *MAT1* loci in selected *Clonostachys* species. The *MAT1* locus structure supports heterothallism in all species, where only either the *MAT1‐1* or *MAT1‐2* idiomorph is present per strain. For both *C. rhizophaga* and *C. chloroleuca*, both *MAT1‐1* and *MAT1‐2* strains were present, further supporting heterothallism as the sexual strategy in these species. (C) Synteny maps of contigs harbouring *mat* genes in selected homothallic *C. rosea* strains. Only two of the *C. rosea* homothallic strains harboured almost fully intact loci and both are illustrated here. The remaining nine homothallic strains had *mat* genes that were scattered throughout the genome assembly on much smaller contigs, likely due to assembly fragmentation caused by repetitive sequences within the expanded locus. This is represented here by strain CBS 115883 but is true for all nine isolates. In all panels, gene orientation is indicated by arrow direction.

Based on sequence similarity analyses, *mat* genes were identified in all 79 *Clonostachys* genomes, typically found within complete *MAT1* loci and flanked by the *apn2* and *sla2* genes (Figure 1A,B, Table S5 in Supporting Information S1). Nineteen of the *C. rosea* strains harboured typical *MAT1‐1* loci, including the *mat1‐1‐1*, *mat1‐1‐2*, and *mat1‐1‐3* genes. A further 36 *C. rosea* strains harboured typical *MAT1‐2* loci, possessing the *mat1‐2‐1* gene as well as what appears to be a homologue of the *Fusarium mat1‐2‐9* gene. Additional small genes were also identified in several of the *MAT1* loci, some of them with similarity in both *MAT1‐1* and *MAT1‐2* idiomorphs. These were considered annotation artefacts given their presence/absence patterns, presence within both idiomorphs, and short lengths. The five *mat* genes found in *C. rosea* (*mat1‐1‐1*, *mat1‐1‐2*, *mat1‐1‐3*, *mat1‐2‐1* and *mat1‐2‐9*) were also found in *C. chloroleuca*, *C. farinosa* , *C. rhizophaga*, *C. solani* , and *C*. sp. CBS 192.96, always in separate *MAT1‐1* or *MAT1‐2* idiomorphs in each strain (Figure 1B, Table S5 in Supporting Information S1). Presence of either the *MAT1‐1* or *MAT1‐2* idiomorph in these strains suggests heterothallism in most *Clonostachys* species.

The remaining 11 *C. rosea* strains possessed all five *mat* genes, representing both the *MAT1‐1* and *MAT1‐2* idiomorphs, suggesting homothallism. Interestingly, these genes were found in greatly expanded *MAT1* loci of more than 40 kb. In contrast, the *MAT1‐1* and *MAT1‐2* idiomorphs from the heterothallic strains ranged from 12 to 18 kb in length, from *apn2* to *sla2* (Figure 1A). Notably, of the 11 genomes from homothallic strains, only CBS 216.74 and CBS 438.70 had almost fully assembled *MAT1* loci, with all five *mat* genes on a single contig. In the other nine homothallic strains, the genes were present on much shorter contigs, typically with the *mat1‐1‐1* and *mat1‐1‐2* genes on a single contig, *mat1‐1‐3* on a single contig, and *mat1‐2‐1* and *mat1‐2‐9* on a single contig (Figure 1C). This is likely the result of the *mat* genes in these strains being interspersed by AT‐rich, highly repetitive DNA. This sequence type is typically very difficult to assemble into contiguous sequence.

Examination of codon alignments for all five *mat* genes revealed no evidence of premature stop codons or frameshift mutations in the homothallic strains (Supporting Information S2). Selection analyses of *mat1‐2‐1* and *mat1‐2‐9* using *C. rhizophaga* YKD0085 as outgroup detected no significant evidence for relaxed selective constraint in homothallic lineage. HyPhy RELAX detected no significant change in selection intensity for either gene (*mat1‐2‐1*: K = 0.77, *p* = 0.632; *mat1‐2‐9*: K = 1.15, *p* = 0.792), and codeml branch‐model analyses found no significant differences in dN/dS ratios between homothallic and heterothallic lineages (*mat1‐2‐1*: LRT = 0.36, *p* = 0.549; *mat1‐2‐9*: LRT = 1.25, *p* = 0.264).

After 1 month of growth on PDA, there were varying amounts of perithecia, asci, and ascospores produced by the homothallic strains (Supporting Information S3). When homothallic strains were confronted with each other in dual cultures, there was no production of perithecia in the interaction zones after 1 month, showing that perithecia production was not triggered by non‐self‐interaction. Typically, interactions either consisted of a clearing zone between the strains or profuse production of aerial mycelium with no clear sign of hyphal anastomosis (Supporting Information S3). No perithecia were observed in single cultures of any heterothallic strain (data not shown). Dual confrontation of selected heterothallic strains carrying opposite *MAT1* idiomorphs did not result in the production of perithecia on PDA or SC medium after 1 month (Supporting Information S3).

### 3.3 Phylogenetic Relationships of *Clonostachys* Strains

**FIGURE 2:**
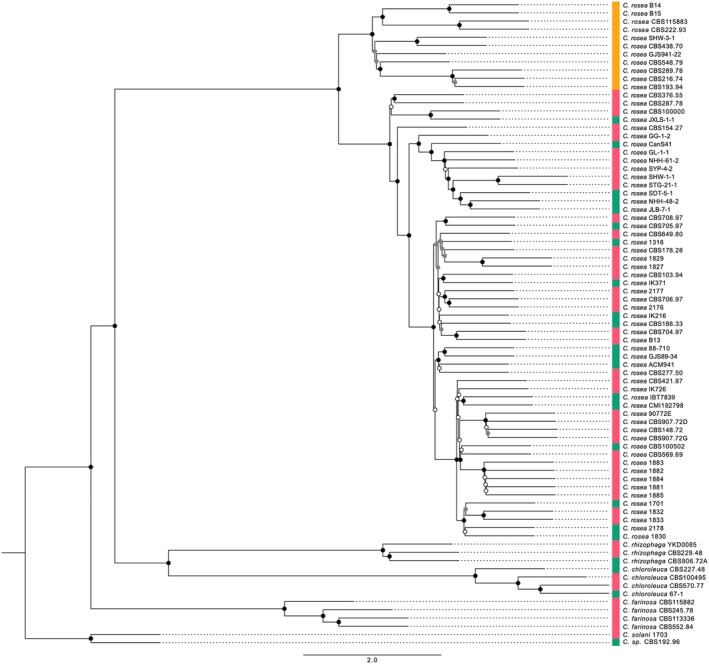
The phylogenetic relationship between homothallic and heterothallic strains reveals a single origin of homothallism in *C. rosea*. The phylogeny is based on 2800 BUSCO proteins present in the genomes of all 66 *C. rosea* strains and additional outgroups. Node support, measured by local posterior probabilities (LPP), are illustrated by black (LPP = 1), grey (0.80 < LPP < 0.99), and white (LPP < 0.8) circles positioned on each node. The track next to each strain represents sexual strategy. Strains harbouring both *MAT1‐1* and *MAT1‐2* idiomorphs, indicating homothallism, annotated in orange. Strains harbouring the *MAT1‐1* idiomorph (annotated in green) or the *MAT1‐2* idiomorph (annotated in pink) are likely heterothallic. This analysis supports the clustering of the homothallic strains separately from the heterothallic strains.

**FIGURE 3:**
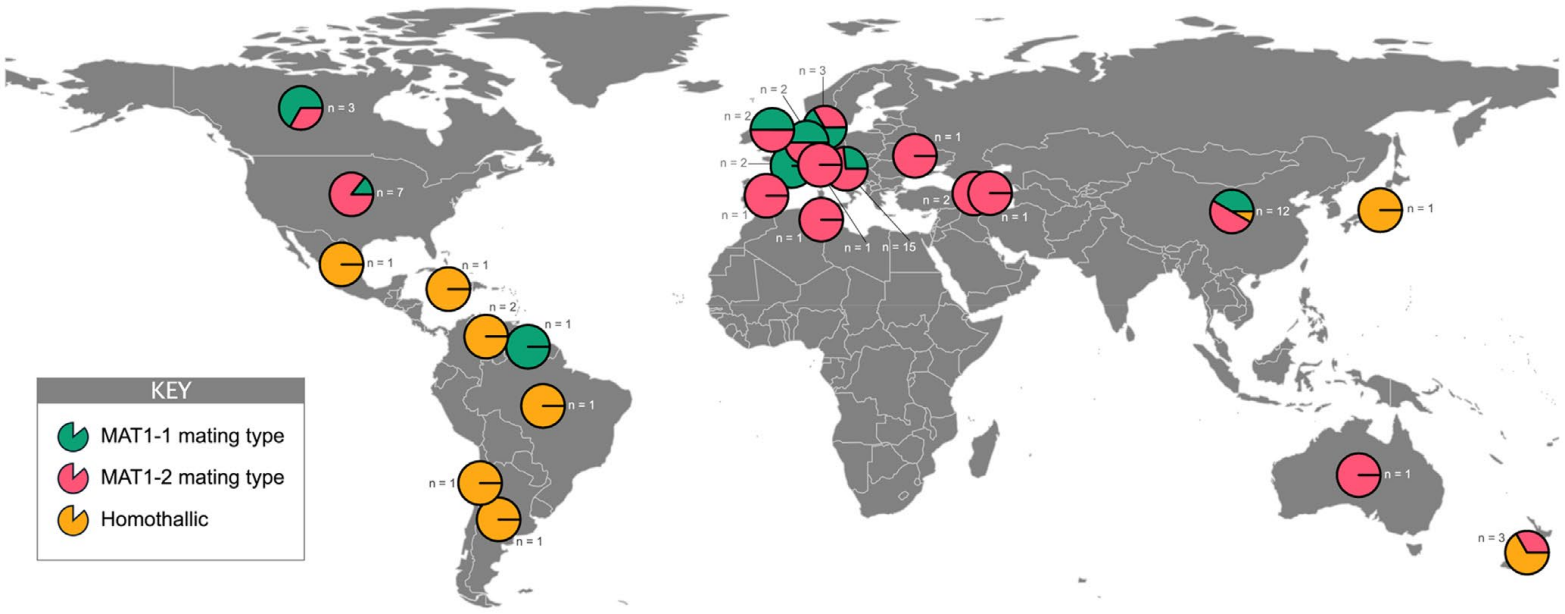
Geographic distribution of heterothallic and homothallic *C. rosea* strains. Homothallic strains, coloured in orange, mainly originate from South and Central America, while heterothallic strains, coloured in green and pink for the MAT1‐1 and MAT1‐2 mating types, respectively, mainly originate from North America, Europe and Asia. This supports the hypothesis of a single origin of homothallism, likely in South America, and subsequent spread to other continents. The number of strains encompassed in each pie‐chart is indicated as *n*.

The phylogenetic analysis of 2800 BUSCO genes clustered heterothallic and homothallic strains into two separate lineages with an LPP value of 1, strongly indicating that these two lineages have diverged (Figure 2). Within the homothallic lineage, one group contained two strains from New Zealand (B14 and B15), one strain from Argentina (CBS 115883) and one strain from Chile (CBS 222.93). Among the remaining strains, SHW‐3‐1 from China and CBS 438.70 from Japan clustered together, while the remaining strains originated from Brazil, Jamaica, Mexico and Venezuela. Among heterothallic strains, some clustering according to geographic origin was evident, as most strains from China and some strains from Slovenia clustered in distinct groups. Mapping mating types and/or sexual strategy geographically showed that heterothallic strains containing *MAT1‐1* or *MAT1‐2* idiomorphs existed within the same areas in North America, Europe and China (Figure 3), suggesting the opportunity for sexual reproduction. Countries with single mating types were typically only represented by a single strain and likely represent a sampling bias rather than a true mating type bias. The homothallic strains were restricted to South and Central America, Japan, China and New Zealand, further supporting the single origin of this lineage and a single origin of homothallism in this species.

### 3.4 Population Structure of *Clonostachys rosea*

**FIGURE 4:**
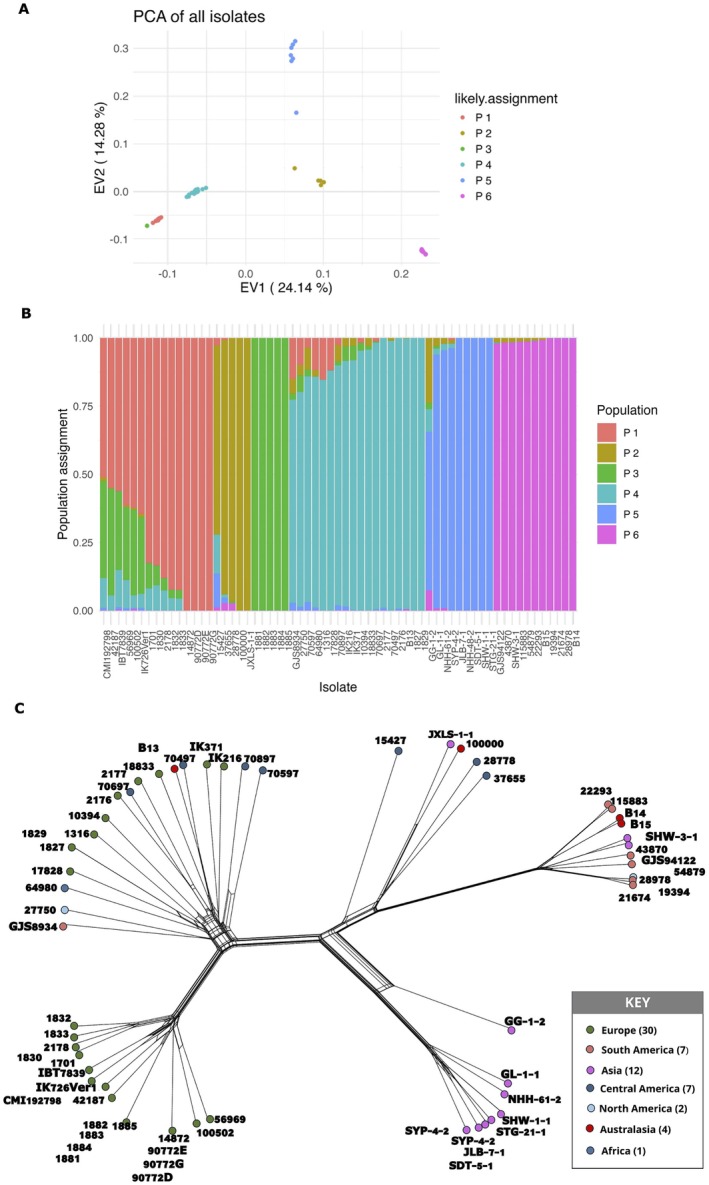
Population structure analyses of heterothallic and homothallic *C. rosea* strains. (A) PCA of population structure of all 63 strains. Eigenvector 1 (24.14% of variance) is plotted on the x‐axis and eigenvector 2 (14.28% of variance) is plotted on the y axis. Points are coloured according to the population to which the corresponding strains have been assigned. (B) Population structure plot of all 63 strains, with each vertical bar representing a strain coloured according to the population to which it has been assigned. (C) Phylogenetic network showing the relationship among all 63 strains. Strain labels are colour‐coded according to their region of origin.

Principal component analysis and model‐based clustering were performed to investigate population genetic structure among 63 *C. rosea* strains (Table S1 in Supporting Information S1). After LD pruning, 31,132 unlinked SNPs were retained for the analysis. The first two principal components, together explaining 38.4% of the genetic variation, separated the strains into six distinct clusters, with the eleven homothallic strains forming a cohesive group (Figure 4A). This pattern was corroborated by the model‐based clustering method sNMF, which identified *K* = 6 populations based on the minimum cross‐entropy criterion. The 11 homothallic strains were assigned to a single population, whereas the 52 heterothallic strains were distributed across five populations (Figure 4B). Heterothallic populations were mainly structured by geography, with some evidence of admixture. European strains predominated in populations 1, 3, and 4, alongside a minority from America and Canada. Most Chinese strains clustered in population 5, whereas several strains from China, Australia, and America formed population 2 (Figure 4B). Homothallic strains from Central and South America, New Zealand, China, and Japan comprised population 6 (Figure 4B). A neighbour‐joining phylogenetic network supported these patterns and revealed multiple reticulations among strains, suggesting shared polymorphisms across lineages (Figure 4C).

### 3.5 Genetic Diversity of *Clonostachys rosea*

**FIGURE 5:**
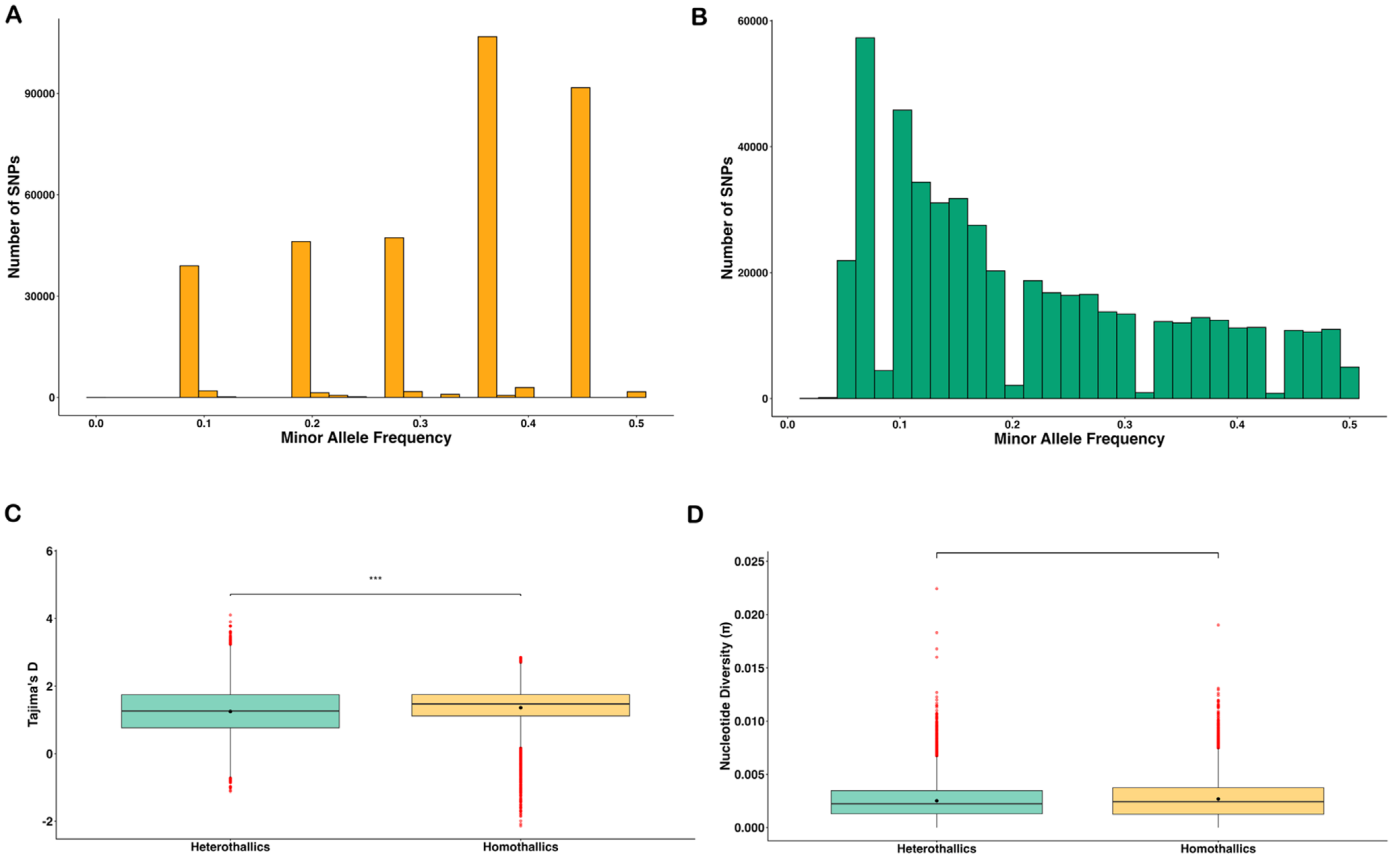
Patterns of genetic diversity in homothallic and heterothallic *C. rosea* strains. (A) Minor allele frequency spectrum for SNPs among homothallic *C. rosea* strains. (B) Minor allele frequency spectrum for SNPs among heterothallic *C. rosea* strains. (C) Comparison of genome‐wide Tajima's D in homothallic and heterothallic groups. Homothallic strains are coloured in orange and heterothallic strains are coloured in green. (D) Comparison of genome‐wide nucleotide diversity (π) in homothallic and heterothallic groups. Homothallic strains are coloured in orange and heterothallic strains are coloured in green.

Genetic diversity was assessed using minor allele frequency spectra, Tajima's D, and nucleotide diversity (π) (Figure 5). A total of 1,018,039 biallelic SNPs were identified across the 63 *C. rosea* strains. After partitioning the whole‐genome SNP dataset by mating type (homothallic versus heterothallic) and applying quality filters, 343,313 high‐confidence SNPs were retained for the homothallic group (11 strains) and 483,898 SNPs for the heterothallic group (52 strains).

In the homothallic group, the minor allele frequency spectrum was enriched for intermediate‐frequency variants, with relatively few low‐ or high‐frequency SNPs (Figure 5A). In contrast, the heterothallic group exhibited a left‐skewed distribution dominated by low‐frequency variants (Figure 5B). Tajima's D was positive in both groups (homothallic mean D = 1.36; heterothallic mean D = 1.25), indicating an excess of intermediate‐frequency SNPs (Figure 5C). A significant difference in mean Tajima's D between groups was supported by bootstrap resampling (*p* < 0.05).

Despite low overall genetic diversity in both groups, homothallic strains showed slightly higher mean genome‐wide nucleotide diversity (π = 0.00269) than heterothallic strains (π = 0.00252); however, this difference was not statistically significant based on bootstrap resampling (*p* > 0.05).

**FIGURE 6:**
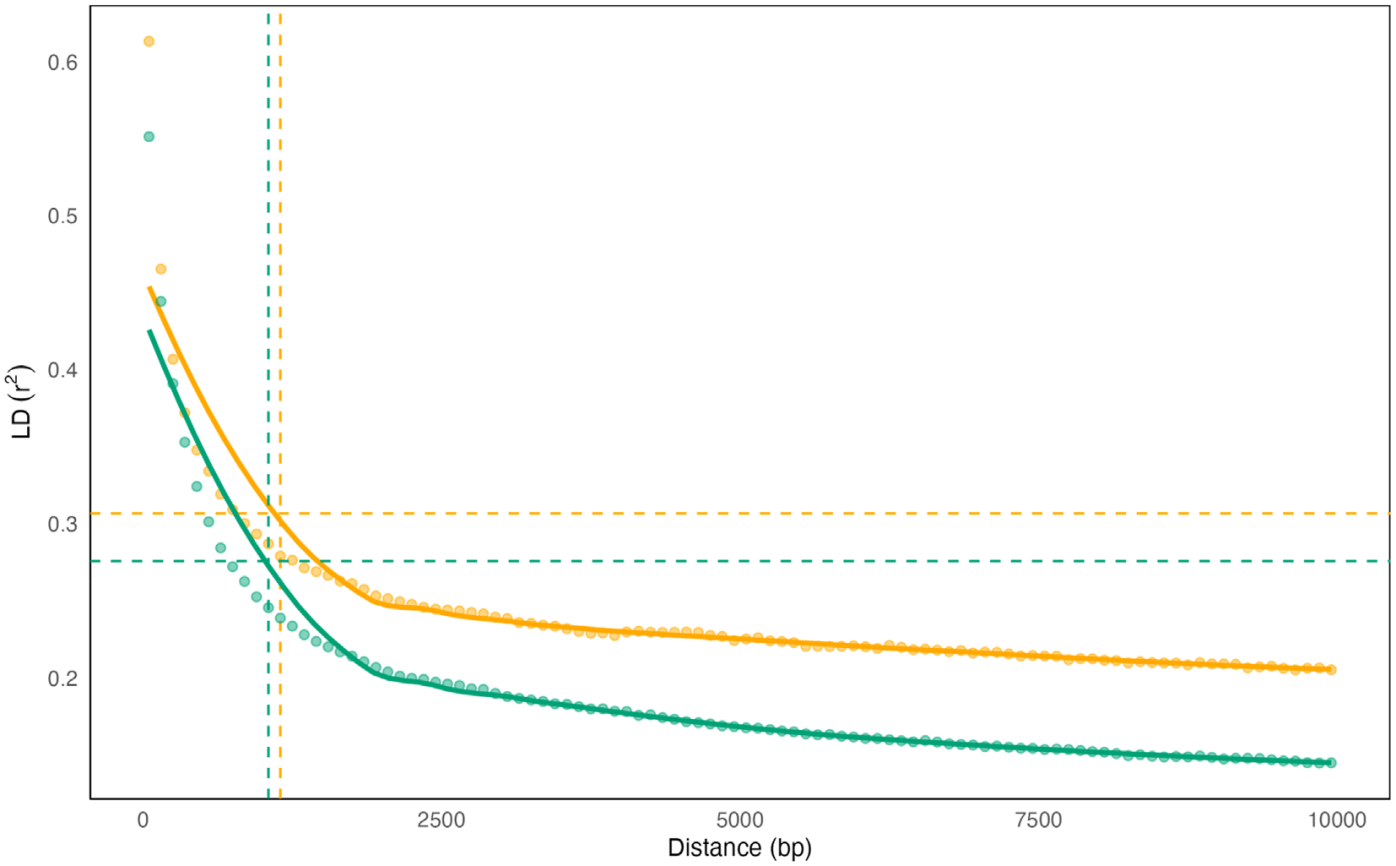
Decay of linkage disequilibrium in homothallic and heterothallic *C. rosea* strains. Linkage disequilibrium (LD) was calculated between pairs of polymorphic sites as a function of the distance between sites in homothallic group, coloured in orange, and heterothallic group, coloured in green. Each point represents the average *r* ^2^ between all pairs of points calculated within sliding 10 kb windows with a step size of 5 kb across the genome. The solid lines are LOESS curves fit to the mean *r* ^2^ values. The vertical dashed lines indicate the position at which LD decayed to half its maximum value in each group. The horizontal dashed lines represent half the maximum LD for each group.

To further characterize recombination history, LD decay was estimated separately for each group. LD declined to half of its maximum value at 1150 bp in homothallic strains and 1050 bp in heterothallic strains (Figure 6).

**FIGURE 7:**
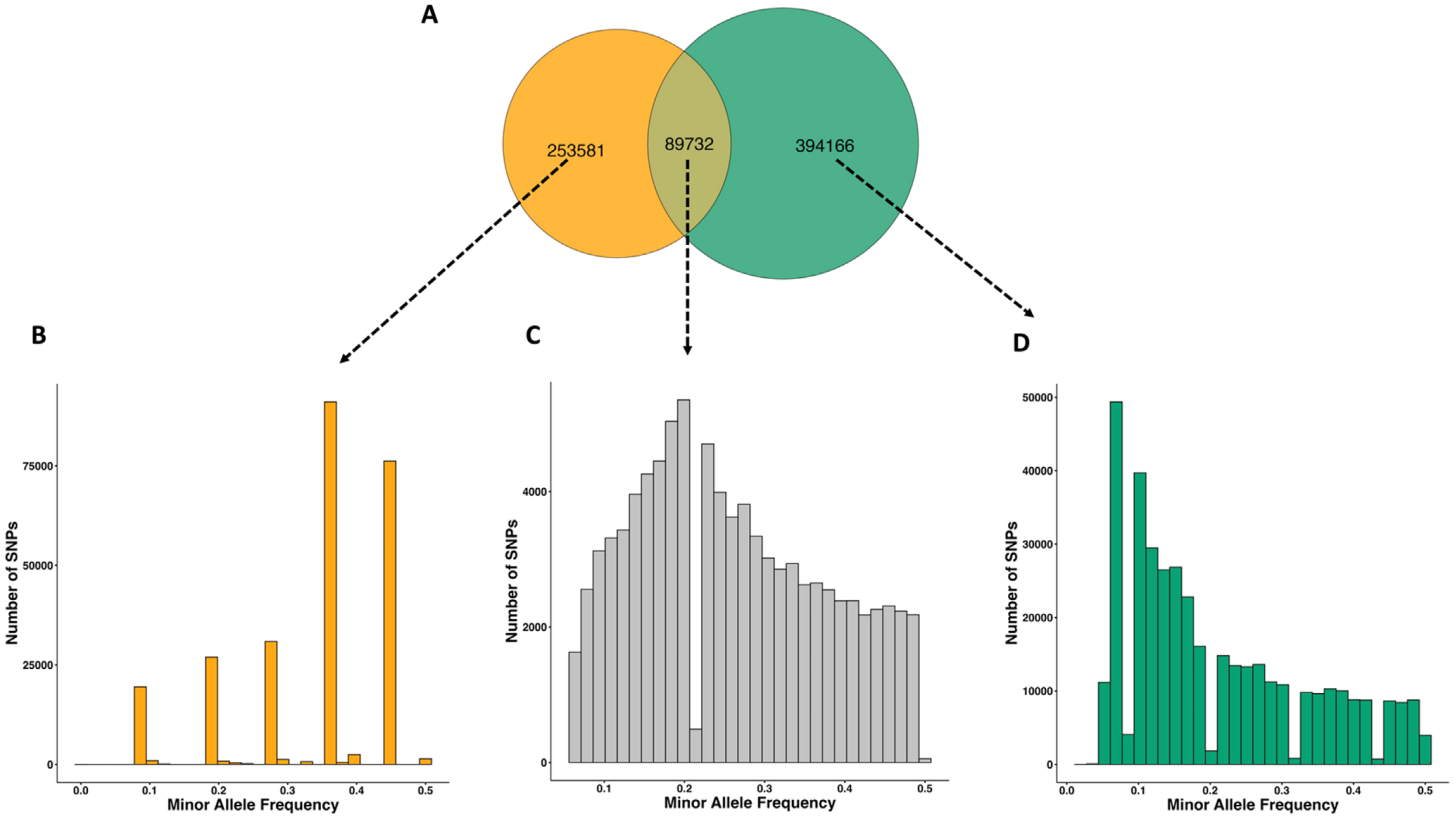
Venn diagram illustrating SNP overlap in homothallic and heterothallic *C. rosea* strains and their corresponding allele frequency spectra. (A) Venn diagram showing the overlap and counts of SNPs in homothallic and heterothallic groups. Minor allele frequency spectrum for (B) the SNPs specific to the homothallic group. (C) the shared SNPs between the homothallic and the heterothallic groups. (D) the SNPs specific to the heterothallic group.

Minor allele frequency distributions were examined for SNPs shared between groups and for SNPs private to each group (Figure 7). SNPs shared between homothallic and heterothallic groups peaked at intermediate allele frequencies (0.2–0.3; Figure 7C), with fewer SNPs at low or high frequencies. Homothallic‐specific SNPs were enriched for intermediate‐frequency variants, with a peak between 0.3 and 0.4 (Figure 7B), whereas heterothallic‐specific SNPs exhibited a left‐skewed spectrum dominated by low‐frequency variants (Figure 7D).

To assess the distribution of functional genetic variation, nucleotide diversity (π) was compared among predicted functional SNP categories (nonsense, missense, synonymous) within shared and group‐specific SNP sets (Figure 7). For predicted nonsense SNPs, nucleotide diversity was highest in the shared SNPs (π = 0.0114), intermediate in homothallic‐specific SNPs (π = 0.0107), and lowest in heterothallic‐specific SNPs (π = 0.0049). Pairwise bootstrap comparisons showed that diversity was significantly higher in homothallic‐specific than in heterothallic‐specific SNPs (*p* < 0.05), higher in shared than in heterothallic‐specific SNPs (*p* < 0.05), and significantly higher in shared than in homothallic‐specific SNPs (*p* < 0.05). For predicted missense SNPs, nucleotide diversity was highest in homothallic‐specific SNPs (π = 0.0079), followed by shared (π = 0.0064), and heterothallic‐specific SNPs (π = 0.0046). Homothallic‐specific SNPs showed significantly higher diversity than heterothallic‐specific SNPs (*p* < 0.05), whereas homothallic‐specific and shared SNPs did not differ significantly (*p* > 0.05). Shared SNPs exhibited significantly higher diversity than heterothallic‐specific SNPs (*p* < 0.05). A similar pattern was observed for predicted synonymous SNPs, with the highest diversity in homothallic‐specific SNPs (π = 0.0076), followed by shared (π = 0.0064) and heterothallic‐specific SNPs (π = 0.0048). Homothallic‐specific and heterothallic‐specific SNPs differed significantly (*p* < 0.05), whereas homothallic‐specific and shared SNPs did not (*p* > 0.05); shared SNPs again showed significantly higher diversity than heterothallic‐specific SNPs (*p* < 0.05).

Finally, genome‐wide pN/pS ratios calculated across all SNPs differed significantly between groups. The homothallic group exhibited lower pN/pS (0.23) than the heterothallic group (0.29) (Welch's *t*‐test *p* < 0.001; Wilcoxon test *p* < 0.001), indicating a lower relative proportion of nonsynonymous to synonymous variants in the homothallic lineage.

## 4 Discussion

We investigated the sexual reproductive mode of *C. rosea* to resolve the contradiction between earlier reports of homothallism (Schroers et al. 1999) and the rapid genome‐wide LD decay indicative of outcrossing (Broberg et al. 2018). Using a globally representative population genomic dataset, genomic analyses identified both homothallic and heterothallic strains based on the structure and gene content of the *MAT1* locus, and phylogenomics supported a single origin of homothallism within *C. rosea* . Population genomic analyses showed that homothallic and heterothallic strains form distinct genetic clusters and differ in multiple population‐genetic parameters. These results reveal a rare situation in which homothallic and heterothallic lineages coexist within a single fungal species, providing an exceptional opportunity to investigate the genomic consequences of selfing and outcrossing in a shared genomic background.

The 53–62 Mb size range of our Illumina‐based *C. rosea* genome assemblies is consistent with previously reported Illumina‐based assemblies, including 58.3 Mb for *C. rosea* IK726 ver. 1 (Karlsson et al. 2015), 56.9 Mb for *C. rosea* ACM941, and 55.5 Mb for *C. rosea* 88‐710 (Demissie et al. 2021). Based on the assumption of homothallism, we expected all *C. rosea* genomes to carry both *MAT1‐1* and *MAT1‐2* idiomorphs, but most strains contained only one of the two *MAT1* idiomorphs, indicating heterothallic reproduction. Presence of the *mat1‐2‐1* gene was previously reported in *C. rosea* strain IK726 (Karlsson et al. 2015) and is confirmed here. All other *Clonostachys* species carried only *MAT1‐1* or *MAT1‐2* idiomorphs, indicating heterothallism as the ancestral state for the genus, with homothallism as a relatively recently derived condition in *C. rosea* . Similar patterns of heterothallism as ancestral and homothallism as derived have been reported in multiple ascomycete lineages, including *Botryosphaeriaceae* (Nagel et al. 2018), *Neurospora* (Gioti et al. 2012), and *Cochliobolus* (Yun et al. 1999). The co‐occurrence of MAT1‐1 and MAT1‐2 strains in North America, Europe, and China, together with short LD decay, suggests the potential for frequent sexual outcrossing and recombination in heterothallic *C. rosea* strains, similar to patterns seen in highly recombining fungal pathogens such as *Zymoseptoria tritici*, where rapid LD decay and balanced mating type ratios indicate regular sexual reproduction (Croll and McDonald 2012; Feurtey et al. 2023). Earlier work documenting substantial local multilocus diversity of *C. rosea* in a Danish field is consistent with this picture of historically high gene flow and recombination (Bulat et al. 2000). Heterothallic strains also show global population genetic structure. While this is partly due to geography, where, for example, most strains from China are genetically similar, there are also strains from three different continents clustering together, a pattern comparable to cosmopolitan plant‐associated fungi such as *Z. tritici* and *F. graminearum* (Croll and McDonald 2012; Feurtey et al. 2023; O'Donnell et al. 2000; Wang et al. 2011). The factors driving this population differentiation remain unknown and require further exploration.

Eleven *C. rosea* strains harboured genes from both *MAT1‐1* and *MAT1‐2* idiomorphs, consistent with homothallism. The localisation of *MAT1‐1* and *MAT1‐2* idiomorph genes in tandem on the same contig in at least two strains suggests that the evolutionary mechanism that allowed for the transition from heterothallism to homothallism is an unequal crossing‐over event that brought together all genes required for selfing. Tandem *MAT1* idiomorphs due to unequal crossing‐over have been reported in several fungi, including *N. pannonica* and *N. terricola* (Gioti et al. 2012), *F. graminearum* (O'Donnell et al. 2004), and *Cochliobolus luttrellii* (Yun et al. 1999), suggesting that it is a common mechanism for transitions from heterothallism to homothallism. Several homothallic strains produced perithecia in single culture, with asci containing ascospores, indicating that the *MAT1* locus structure likely confers homothallism. *Clonostachys rosea* strains originating from ascospores have been reported to frequently produce perithecia in single cultures (Schroers et al. 1999). Notably, all strains originally reported as the *B. ochroleuca* teleomorph in our current investigation possessed the homothallic *MAT1* locus.

Phylogenomic analysis shows that all homothallic strains form a well‐supported monophyletic lineage, reinforced by population structure analyses, indicating descent from a single unequal crossing‐over event between two distinct heterothallic ancestors. The lack of observed anastomosis between homothallic strains suggests they have accumulated enough genetic variation to be recognized as non‐self in vegetative interactions. We hypothesize that this transition occurred in South America, where most homothallic strains were collected. This is further supported by the phylogenetic structure among homothallic strains where a clear distinction can be seen between strains from the south versus the north parts of South America. Under this scenario, the homothallic lineage may have first spread within South America and subsequently into Central America and the Caribbean. Furthermore, this scenario suggests at least two independent inter‐continental movements of homothallic *C. rosea* : one from southern South America to New Zealand, and another from northern South America/Central America to Japan and China. These movements may be anthropogenic, as in other plant‐ and soil‐associated fungi, including *Z*. *tritici* and *Heterobasidion annosum*, where dispersal is linked to infected plant material or soil (Croll and McDonald 2012; Dalman et al. 2010; Feurtey et al. 2023). Given the endophytic and soil‐associated lifestyle of *C. rosea* (Mendoza García et al. 2003; Mueller and Sinclair 1986; Nobre et al. 2005; Walker and Maude 1975), movement of plants and associated soil provides a plausible mechanism for this inter‐continental spread.

The successful geographic expansion suggests that the homothallic lineage has high ecological fitness despite the potential long‐term costs of selfing, though the exact fitness benefit remains unknown. One possibility is that homothallism preserved a favourable combination of alleles, as proposed for other selfing or weakly recombining lineages of plant‐associates, such as *Phytophthora infestans* and *Epichloe* endophytes, where particular genotypes remain successful despite reduced recombination (Drenth et al. 2019; López‐Villavicencio et al. 2013; McDonald and Linde 2002; Schardl et al. 2013). At the same time, comparative analyses of fungal mating systems indicate that transitions from heterothallism to homothallism are often unidirectional, with selfing lineages frequently viewed as evolutionary “dead ends” that rarely revert to outcrossing (Gioti et al. 2012). Classical theory predicts that obligate selfing promotes accumulation of slightly deleterious mutations through Muller's ratchet, reducing fitness and increasing extinction risk (Felsenstein 1974; Glémin and Galtier 2012; Muller 1932, 1964). The coexistence of homothallic and heterothallic lineages in *C. rosea* therefore provides a particularly interesting case to study how short‐term benefits and long‐term risks of selfing manifest at the genomic level.

The structure of the *MAT1* locus suggests mutation accumulation in homothallic strains. Heterothallic strains have highly conserved *MAT1* idiomorphs, whereas homothallic *MAT1* loci contain extensive AT‐rich repetitive DNA. These repetitive regions prevented full‐length assembly of the *MAT1* locus in many homothallic strains, resulting in *mat* genes being located on short, fragmented contigs. Similar difficulties in assembling *MAT1* have been reported in other fungi, such as *Sclerotinia borealis*, where repeat‐rich *MAT1* regions complicate reconstruction of idiomorph structure (Wilson et al. 2023). In homothallic *Neurospora* species, *mat* genes are reported to evolve under relaxed constraint and accumulate frameshift and stop codon mutations (Wik et al. 2008). However, our analyses found no evidence of premature stop codons, frameshift mutations, or relaxed selection in the *mat* genes of the homothallic *C. rosea* strains. Furthermore, several homothallic *C. rosea* strains produced perithecia, as well as asci with ascospores, despite extensive amounts of repetitive DNA in their *MAT1* locus, indicating that the *mat* genes remain functional in these strains. One possible explanation is that the homothallic lineage originated relatively recently, leaving insufficient time for substantial degeneration to accumulate.

On a genome‐wide level, homothallic strains showed higher nucleotide diversity than heterothallic strains within the predicted nonsense, missense, and synonymous lineage‐specific SNP classes. This pattern is consistent with mutation accumulation under reduced effective recombination (Glémin and Galtier 2012), although demographic history may also contribute. The high nucleotide diversity of shared nonsense SNPs likely reflects ancestral polymorphisms maintained at intermediate frequencies, whereas the lower nucleotide diversity of heterothallic‐specific SNPs may reflect more efficient purging of deleterious variants and an excess of recent low‐frequency mutations in the larger heterothallic metapopulation. Similar effects of demographic history on nucleotide diversity have been documented in rapidly spreading fungal pathogens (Charlesworth and Wright 2001; Talas and McDonald 2015).

The lower genome‐wide pN/pS ratios observed in homothallic strains are consistent with expectations under the nearly neutral model of molecular evolution (Charlesworth and Eyre‐Walker 2008; Eyre‐Walker and Keightley 2007). Because pN/pS estimates based on standing polymorphism are sensitive to allele‐frequency distributions, the excess of low‐frequency mildly deleterious nonsynonymous variants in the large structured heterothallic metapopulation likely contributes to the elevated pN/pS observed in heterothallic strains. In contrast, the homothallic lineage appears recently derived from a recombining ancestor, retains substantial ancestral polymorphism, and shows fewer rare variants, consistent with limited time for deleterious non‐synonymous variants to accumulate. Similar patterns have been reported in recently derived plant selfers such as *Capsella rubella* (Brandvain et al. 2013; Slotte et al. 2013).

A critical question is whether the homothallic *C. rosea* lineage is obligately homothallic or occasionally outcrosses. Elevated Tajima's D values and enrichment for intermediate‐frequency alleles are consistent with limited recombination in homothallic strains (Charlesworth and Wright 2001; Tajima 1989), whereas the excess of low‐frequency variants in heterothallic strains is typical of recombining populations (Croll and McDonald 2012; Feurtey et al. 2023). The intermediate frequencies of shared SNPs suggest that many variants segregated in the ancestral heterothallic population before the origin of homothallism. Likewise, the enrichment of intermediate‐frequency homothallic‐specific SNPs is consistent with recent selfing and founder effects following the establishment of the homothallic lineage (Brandvain et al. 2013; Charlesworth 2003; Wakeley and Aliacar 2001).

Additional insight into the extent of recombination within the homothallic lineage comes from patterns of LD decay, a standard measure of recombination (Slatkin 2008), which was similarly short in homothallic (1150 bp) and heterothallic (1050 bp) lineages, comparable to values reported for highly recombining fungi such as *F. graminearum* and *Z. tritici* (approximately 164–2 kb) (Croll and McDonald 2012; Feurtey et al. 2023; Singh et al. 2021; Talas and McDonald 2015) The slightly longer LD in the homothallic strains is consistent with reduced effective recombination under selfing, although LD is also influenced by demography and effective population size (Charlesworth and Wright 2001; Glémin and Galtier 2012). The small difference between lineages suggests either occasional outcrossing in the homothallic lineage, as reported for other homothallic fungi such as *A. nidulans* (López‐Villavicencio et al. 2013), or a recent origin of homothallism, such that historical recombination in the ancestral heterothallic population continues to shape present‐day LD patterns. Theoretical and empirical work shows that genomic signatures of maladaptation in selfing lineages can be subtle and are easily obscured by low levels of residual outcrossing or recent transitions to selfing (Escobar et al. 2010; Glémin and Galtier 2012; Loewe and Cutter 2008). While the homothallic lineage is represented by a limited number of strains (*n* = 11), the interpretation of a recent transition to selfing is supported by congruent evidence from *MAT1* locus structure, phylogenomics, and multiple population genetic analyses. Nevertheless, additional sampling of homothallic populations will be important for further testing these hypotheses.

Occasional outcrossing is relevant to whether the homothallic *C. rosea* lineage should be regarded as a separate species. Under lineage‐based species concepts, species are independently evolving lineages (de Queiroz 1998), and an obligate homothallic lineage could in principle be viewed as a separate species. Although it is clearly differentiated in genome‐wide phylogenies and mating‐type organization, shared polymorphisms, detectable admixture, and reticulation in the neighbour‐net indicate that complete reproductive isolation has not evolved. We therefore view the homothallic lineage as a recently derived lineage that is still diverging from heterothallic *C. rosea* rather than a fully separated species.

Population genomic datasets have been used extensively to dissect agriculturally important traits in fungi through genome‐wide association studies (GWAS), including virulence in pathogens such as *H. annosum* (Dalman et al. 2013), *Z. tritici* (Gao et al. 2016), *Rhynchosporium commune* (Mohd‐Assaad et al. 2016), and *F. graminearum* (Talas and McDonald 2015). As some *C. rosea* strains, such as the heterothallic ACM941 and IK726, have attracted commercial interest as biological control agents (Jensen et al. 2022), similar approaches could identify genetic determinants of biocontrol efficacy for marker development. The identification of distinct homothallic and heterothallic lineages also has practical implications for GWAS in *C. rosea* . Because homothallic strains likely experience reduced effective recombination and different linkage structures, including them in association panels dominated by heterothallic strains may violate key GWAS assumptions (Croll and McDonald 2012). Previous GWAS in *C. rosea* investigated growth at cold temperature (Broberg et al. 2018) and production of nematicidal compounds (Iqbal et al. 2020). However, only a single homothallic strain (CBS 193.94) was included in these studies and is therefore unlikely to have substantially affected the results. Based on the current work, we recommend that future GWAS exclude homothallic strains.

In summary, we identified coexisting homothallic and heterothallic lineages within *C. rosea* and show that homothallism likely originated once through *MAT1* locus restructuring. Our results reconcile the apparent contradiction between previously reported homothallism and rapid LD decay, inferring a recent origin and successful geographic expansion of a homothallic lineage. Our data provide an important framework for future population genomic and association studies aiming to identify the genetic basis for biocontrol‐related traits in *C. rosea* .

## Author Contributions

D.M., A.M.W., M.D., M.B.D. and M.K. planned and designed the experiments. B.J., A.R. and M.D. performed the lab work, while D.M., A.M.W., E.P., M.B.D., M.B., S.C., H.D.F.L., M.D. and M.K. analysed the data. All authors read and approved the manuscript.

## Funding

This work was supported by the Swedish Research Council for Environment, Agricultural Sciences and Spatial Planning (FORMAS) (grant number 942‐2015‐1128), by the Department of Forest Mycology and Plant Pathology at the Swedish University of Agricultural Sciences, and by SLU Grogrund.

## Ethics Statement

The authors have nothing to report.

## Conflicts of Interest

The authors declare no conflicts of interest.

## Supporting information


**Data S1:** Supporting Information.
**Table S1:** Clonostachys rosea strains used in the current study.
**Table S2:** RNA‐seq datasets used for gene prediction in the current study.
**Table S3:** Genomes from additional species and strains used in the current study.
**Table S4:** Genome statistics for Clonostachys rosea genomes generated in the current study.
**Table S5:** Distribution of mating type idiomorphs in Clonostachys strains.

**Data S2:** Supporting Information.
**Dataset:** S1. Fasta‐format alignment of Clonostachys rosea mat1‐1‐1 genes.
**Dataset:** S2. Fasta‐format alignment of Clonostachys rosea mat1‐1‐2 genes.
**Dataset:** S3. Fasta‐format alignment of Clonostachys rosea mat1‐1‐3 genes.
**Dataset:** S4. Fasta‐format alignment of Clonostachys rosea mat1‐2‐1 genes.
**Dataset:** S5. Fasta‐format alignment of Clonostachys rosea mat1‐2‐9 genes.

**Data S3:** Supporting Information.
**Figure S1:** Phenotypic analyses of Clonostachys rosea strains. Homothallic C. rosea strains were individually grown on PDA and incubated at 20°C in darkness. After 2 weeks, perithecium formation was visible and examined daily for the following 2 weeks. (A, B) Macroscopic view of perithecia formation in C. rosea strain CBS 115883. (C, D) Perithecia formation in C. rosea strain B15. (E) Release of asci from perithecia of C. rosea strain CBS 289.78. (F) Combinations of different homothallic C. rosea strains were inoculated on PDA and incubated at 20°C in darkness to monitor the interaction behaviour. (G) Combinations of different heterothallic C. rosea strains were inoculated on PDA and SC (crossing) agar medium and incubated at 20°C in darkness to monitor the interaction behaviour.

## Data Availability

Raw sequencing reads and genome assemblies are available from the European Nucleotide Archive (ENA) under Bioproject PRJEB108086.
