# Supplementary material for: Homothallic or Heterothallic? A Genomic Investigation Into the Sexual Capabilities of the Ascomycete Fungus Clonostachys rosea
Source: Mol Ecol. 2026 Jul 25;35(14):e70486. doi: 10.1111/mec.70486 (PMC13401452; doi:10.1111/mec.70486)
Supplement: Supplementary file 3 — Data S3: Supporting Information. Figure S1: Phenotypic analyses of Clonostachys rosea strains. Homothallic C. rosea strains were individually grown on PDA and incubated at 20°C in darkness. After 2 weeks, perithecium formation was visible and examined daily for the following 2 weeks. (A, B) Macroscopic view of perithecia formation in C. rosea strain CBS 115883. (C, D) Perithecia formation in C. rosea strain B15. (E) Release of asci from perithecia of C. rosea strain CBS 289.78. (F) Combinations of different homothallic C. rosea strains were inoculated on PDA and incubated at 20°C in darkness to monitor the interaction behaviour. (G) Combinations of different heterothallic C. rosea strains were inoculated on PDA and SC (crossing) agar medium and incubated at 20°C in darkness to monitor the interaction behaviour. [file MEC-35-e70486-s003.pdf]

Supporting Information S3, Figure S1

Examples of perithecia and ascospore production in homothallic *C. rosea* strains.

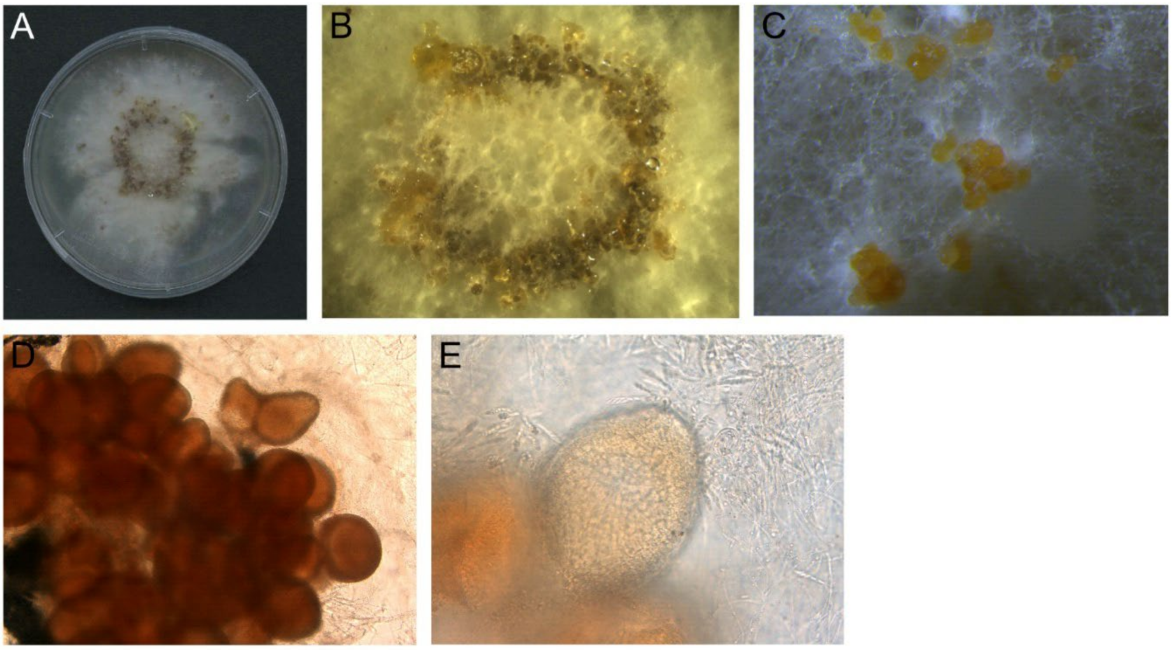

Examples of dual confrontations between homothallic *C. rosea* strains.

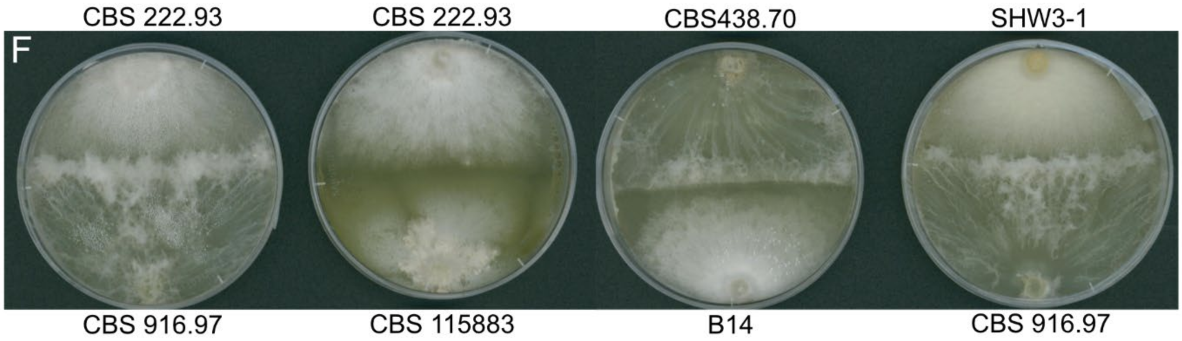

Examples of dual confrontations between heterothallic *C. rosea* strains.

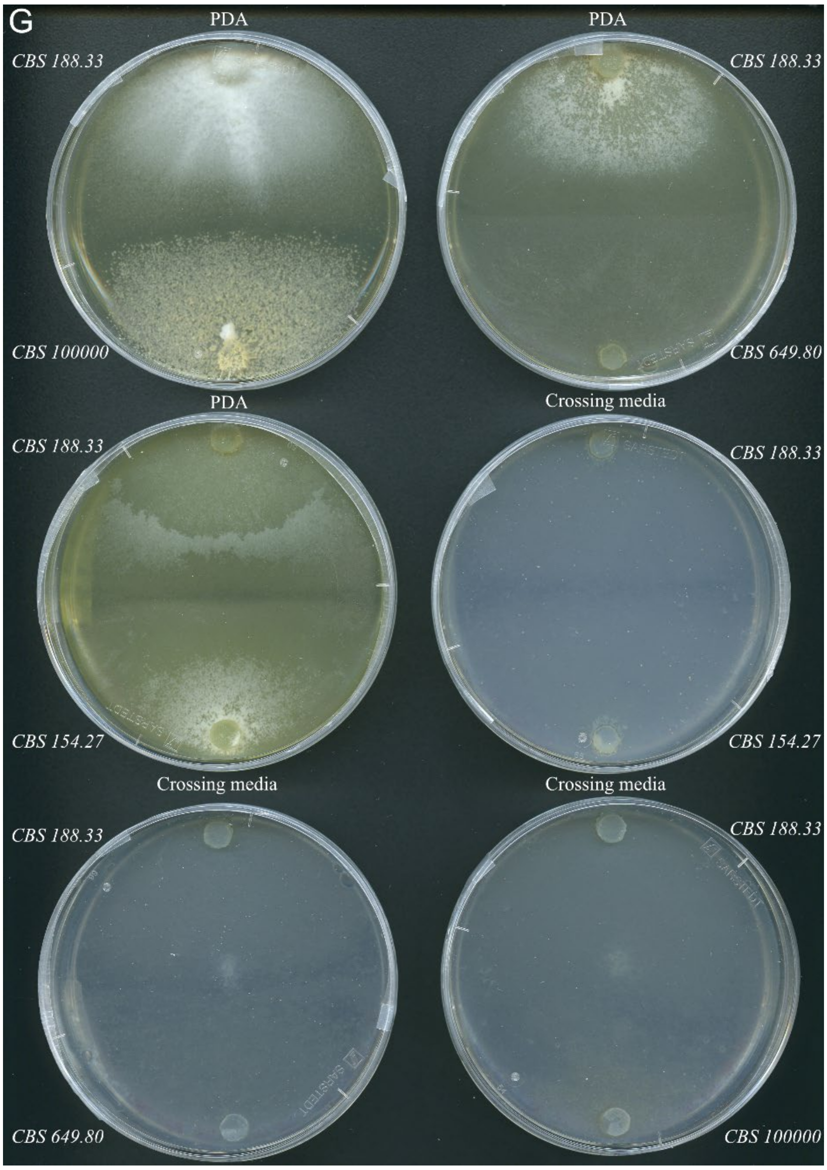

Figure S1. Phenotypic analyses of *C. rosea* strains. Homothallic *C. rosea* strains were individually grown on PDA and incubated at 20°C in darkness. After two weeks, perithecium formation was visible and examined daily for the following 2 weeks. (**A, B**) Macroscopic view of perithecia formation in *C. rosea* strain CBS 115883. (**C, D**) Perithecia formation in *C. rosea* strain B15. (**E**) Release of asci from perithecia of *C. rosea* strain CBS 289.78. (**F**) Combinations of different homothallic *C. rosea* strains were inoculated on PDA and incubated at 20°C in darkness to monitor the interaction behaviour. (**G**) Combinations of different heterothallic *C. rosea* strains were inoculated on PDA and SC (crossing) agar medium and incubated at 20°C in darkness to monitor the interaction behaviour.
